# Expression of *Bordetella pertussis* Antigens Fused to Different Vectors and Their Effectiveness as Vaccines

**DOI:** 10.3390/vaccines9060542

**Published:** 2021-05-21

**Authors:** Han Xu, Jing Huang, Zhaolu Liu, Xin Li, Kangfeng Wang, Erling Feng, Jun Wu, Li Zhu, Kaihu Yao, Chao Pan, Hengliang Wang

**Affiliations:** 1 State Key Laboratory of Pathogen and Biosecurity, Beijing Institute of Biotechnology, 20 Dongdajie Street, Fengtai District, Beijing 100071, China; Hanx15666259216@163.com (H.X.); jinghuangpaper0203@163.com (J.H.); 13173224823@163.com (Z.L.); xinli0531@163.com (X.L.); wangkf1220@126.com (K.W.); fengel@sohu.com (E.F.); junwu1969@163.com (J.W.); jewly54@126.com (L.Z.); 2 Beijing Children’s Hospital Affiliated to Capital University of Medical Sciences, Nan Lishi Road, Xicheng District, Beijing 100045, China; yaokaihu@bch.com.cn

**Keywords:** pertussis, *Bordetella pertussis*, fimbriae, pertussis toxin, filamentous hemagglutinin, fusion antigen, immunological evaluation

## Abstract

Pertussis is an acute respiratory tract infection caused by *Bordetella pertussis*. Even though its current vaccine coverage is relatively broad, they still have some shortcomings such as short protection time and might be incapable of blocking the spread of the disease. In this study, we developed new pertussis vaccine candidates by separately fusing three pertussis antigens (*B. pertussis* fimbriae 2 “Fim2”, pertussis toxin S1 subunit “PtxS1”, and filamentous hemagglutinin “FHA_1877–2250_”) to each of two immune-boosting carrier proteins (B subunits of AB5 toxin family: cholera toxin B subunit “CTB” and shiga toxin B subunit “StxB”). We then immunized mice with these fusion antigens and found that they significantly increased the serum antibody titers and elicited high bactericidal activity against *B. pertussis*. After CTB-or StxB-fused antigen-immunized mice were challenged with a non-lethal dose of *B. pertussis*, the bacterial loads in different tissues of these mice were significantly reduced, and their lung damage was nearly invisible. Furthermore, we also demonstrated that these candidate vaccines could provide strong prophylactic effects against a lethal challenge with *B. pertussis*. Overall, our candidate vaccines conferred better immune protection to mice compared with pertussis antigen alone. This B5 subunit-based vaccine strategy provides a promising option for vaccine design.

## 1. Introduction

Pertussis is a serious respiratory disease that is mainly characterized by persistent and paroxysmal coughing, with a characteristic inspiratory “whooping” sound. Because a typical infection generally lasts for three months, pertussis is also known as the “hundred-day cough” [1]. This disease is caused by *B. pertussis*, an organism mainly spread via aerosols and able to colonize the respiratory tract, where it damages epithelial cells and impairs normal respiratory function. Epidemiological surveys have shown that pertussis outbreaks generally occur every 3–5 years [2]. In the past decade, the number of reported cases has risen to 20,000 to 40,000 confirmed and presumed cases a year [3].

Vaccination is currently the most economical and effective method for preventing pertussis. In the middle of the 20th century, the United States pioneered the development of inactivated whole-cell pertussis vaccine (wPVs), which significant decreased the number of reported pertussis cases. In the 1980s, a safer acellular pertussis vaccine (aPVs) was developed in Japan [4]. At present, aPVs that consist of purified *B. pertussis* antigens are widely used. These antigens include fimbriae (Fim), filamentous hemagglutinin (FHA), pertussis toxin (PT), and pertactin (PRN), which all play roles in bacterial adhesion and infection [5]. Fim, expressed on bacterial surfaces, is a *B. pertussis* pathogenic factor that promotes bacterial binding to host cells. PT is the main virulence factor of *B. pertussis*. It is composed of subunits S1–S5, of which the S1 subunit is the most immunogenic and protective molecule because it contains the major protective epitopes and has ADP-ribosyl transferase activity [6,7]. FHA, involved in the adhesion of bacteria and ciliated epithelium, is another key virulence factor of *B. pertussis*. Research found that amino acid residues 1877–2250 in FHA could invoke the most effective immune response and therefore comprise the most immunogenic site [8]. Although the multi-component composition of aPVs allows them to provide broader coverage, the currently available aPV’s shortcoming is the fading of immunity over the years Consequently, surveys in the many countries have found that the incidence of pertussis is increasing, and several outbreaks have occurred in recent years [5,9,10], known as the “pertussis resurgence”.

Previous studies have revealed that the B subunits in the AB5 toxin family have the potential to enhance immune responses by binding to specific receptors on the surface of cells (particularly antigen-presenting cells). The most widely used one is cholera toxin B subunit (CTB) [11], which acts as a well-known mucosal adjuvant [12,13]. Moreover, both protein and bacterial polysaccharide antigens can be displayed on the CTB via fusion expression or glycosylation, which significantly improves the antigen-specific immune response evoked by the antigens [14,15,16,17]. Another AB5 family member is shiga toxin B subunit (StxB), which is also used as a carrier for immune enhancement. StxB can bind with a specific cell surface receptor (Gb3) that exists on dendritic cells. In addition, StxB can enable exogenous antigens to be recognized by MHC-I in antigen-presenting cells, thereby mediating cellular immune responses [18]. Furthermore, both CTB and StxB are pentamer in nature; this complex structure facilitates antigen delivery. Thus, CTB and StxB are currently considered potential carriers for subunit vaccines in vaccine research and development.

In this study, we separately fused three *B. pertussis* antigens, fimbriae 2 (Fim2), filamentous hemagglutinin amino acids 1877–2250 (FHA_1877–2250_), and pertussis toxin S1 subunit (PtxS1), with CTB or StxB. After mice were subcutaneously immunized with these vaccine candidates, a series of evaluations were conducted, and we found that the immune effects induced by the fusion antigens were significantly higher than those induced by *B. pertussis* antigens alone. Subsequent challenges with either a nonlethal or lethal dose of *B. pertussis* revealed that the vaccine candidates have strong prophylactic effects against *B. pertussis* infection. Therefore, these candidate pertussis vaccines should be examined in further trials against *B. pertussis.* Furthermore, the AB5 subunit-based vaccine strategy may provide a new option for vaccine design.

## 2. Materials and Methods

### 2.1. Strains, Culture Conditions, and Plasmids

*B. pertussis* Bp148 stain was provided by the Beijing Children’s Hospital. Bp148 was cultured at 37 °C in pertussis SS liquid culture or charcoal medium (10 g of peptone, 4 g of charcoal, 5 g of sodium chloride, 0.001 g of niacin, and 12 g of agar dissolved to a final volume of 1 L, to which 20% defibrinated sheep blood was added). The expression strain, BL21 (DE3) competent cells, was purchased from Transgen Biotech Co. Ltd. (Beijing, China) and cultured in Luria–Bertani (LB) broth or LB solid medium (containing 1.5% agar) at 37 °C. To induce protein expression, cells were first cultured in LB broth at 37 °C until the absorbance at 600 nm (OD_600_) reached 0.6, after which isopropyl β-d-1-thiogalactopyranoside (IPTG) was added to a final concentration of 1 mM, and the culture was grown for another 10 h at 30 °C. Kanamycin (50 µg/mL) was used for plasmid selection when required.

### 2.2. Construction of Expression Vectors

The sequences of Fim2, PtxS1, and FHA_1877–2250_, searched from the genome of *Tohama I*, were codon-optimized and synthesized by Detai Biotechnology Co., Ltd. (Nanjing, China). Then the following upstream and downstream primers were used to amplify the Fim2, PtxS1, and FHA_1877–2250_ fragments from the synthetic sequences (Figure S1): Fim2-F and Fim2-R (5′-GAAGGCCTGATGATGGTACCATCGTG-3′ and 5′-CCAAGCTTCGGATAAACACGCTAAA-3′), PtxS1-F and PtxS1-R (5′-CCCATATGGATGATCCGCCGGCGAC-3′ and 5′-CCAAGCTTGAAGCTGTACGCAATGCT-3′), and FHA_1877–2250_-F and FHA_1877–2250_-R (5′-CCCATATGGACGAGCACCGTCACCTG-3′ and 5′-CCAAGCTTACCATCCGCCAGGCTGGT-3′), respectively. Then we obtained the fragments of CTB-Fim2, StxB-Fim2, CTB-PtxS1, StxB-Ptxs1, CTB-FHA_1877–2250_, and StxB-FHA_1877–2250_ by applying whole gene synthesis. The five fragments (CTB-Fim2, PtxS1, FHA_1877–2250_, CTB-FHA_1877–2250_, and StxB-FHA_1877–2250_) were digested with *Nde*I and *Hind*III and then connected to the correspondingly digested pET-30a plasmid. To increase the protein expression efficiency, four other pertussis antigen fragments (Fim2, StxB-Fim2, CTB-PtxS1, and StxB-PtxS1) were digested with *Stu*I and *Hind*III and then connected to the correspondingly digested pSUMO plasmid. Therefore, we obtained three expression vectors (pET-Fim2-SUMO, pET-FHA_1877–2250_, and pET-PtxS1) that expressed the Fim2, FHA_1877–2250_, and PtxS1 antigens, respectively, and six expression vectors (pET-CTB-Fim2, pET-StxB-Fim2-SUMO, pET-CTB-FHA_1877–2250_, pET-StxB-FHA_1877–2250_, pET-CTB-PtxS1-SUMO, and pET-StxB-PtxS1-SUMO) that expressed the six fusion antigens (CTB-Fim2, StxB-Fim2, CTB-FHA_1877–2250_, StxB-FHA_1877–2250_, CTB-PtxS1, and StxB-PtxS1). Among them, Fim2, StxB-Fim2, CTB-PtxS1, and StxB-PtxS1 each had both a 6× His tag and a SUMO tag, whereas the other five fragments had only a 6× His tag at the C-terminal end of the sequence.

### 2.3. SDS-PAGE

Induced cells were harvested via centrifugation at 8000× *g* for 10 min, dissolved in 1× SDS (50 mM Tris-HCl (pH 6.8), 1.6% (*w*/*v*) SDS, 0.02% (*w*/*v*) bromophenol blue dye, and 8% (*v*/*v*) glycerol, 20 mM DL-dithiothreitol), and then incubated in a boiling water bath for 10 min. After the samples were cooled to room temperature, 10-µL samples were collected and used for sodium dodecyl sulfate polyacrylamide gel electrophoresis (SDS-PAGE) as described in our previous study [16].

### 2.4. Protein Purification

After recombinant protein expression was induced, the cells were harvested via centrifugation at 8000× *g* for 10 min, resuspended in A1 solution (0.5 M NaCl, 13.3 mL of Tris-HCl, 8 M urea, and 10 mM imidazole, pH 7.5), and subjected to high-pressure homogenization followed by centrifugation. We then collected the resulting sediment and dissolved it in A1 solution; this suspension was centrifuged at 8000× *g* for 10 min at 4 °C. Finally, the resulting supernatant was collected for purification.

Ten column volumes of A1 solution were used to equilibrate the nickel column, after which the protein sample was loaded at a flow rate of 4 mL/min before A1 solution was used to wash away unbound protein until the ultraviolet absorbance (280 nm) was close to 0 mAU. Finally, five column volumes of buffer B1 (0.5 M NaCl, 13.3 mL of Tris-HCl, 8 M urea, and 0.5 M imidazole, pH 7.5) were used for elution and collection of the target protein. The eluted protein was then placed in a dialysis bag and incubated at 4 °C for 3 days for renaturation. During this period, the urea concentration was continuously decreased, and the protein was finally dialyzed in 1×phostphate-buffered saline (PBS) (pH 8.0) solution. After renaturation, samples were filtered using a 0.22-µM filter and aliquoted before freezing and storage at −80 °C. After purification, a SUMO-specific protease was used to cleave the 6×His tag and SUMO tag (if present) of the proteins.

### 2.5. Animal Immunization Experiments

Five-week-old, specific pathogen-free female BALB/c mice were purchased from SPF Biotechnology (Beijing, China) and reared in the Laboratory Animal Centre of the Academy of Military Medical Sciences. Mice were housed with a constant ambient temperature (23 ± 3 °C) and humidity (55 ± 5%). Food, bedding, and water were changed every 4 days. All animal experiments were approval by the Ethics Committee of the Academy of Military Medical Sciences. Subcutaneous immunization was conducted on Days 0, 14, and 28 in mice. The immunization dose was 100 µL (2 µg of pertussis antigen and 100 µg of aluminum hydroxide adjuvant (General Chemical Corp, Brighton, MI, USA) per mouse, and the control group was immunized with an equal volume of PBS (Table S1). Ten days after the last immunization, blood was collected to test for antibody potency. A bacterial challenge was performed 14 days after the third immunization to evaluate the vaccine-induced protection.

### 2.6. Enzyme-Linked Immunosorbent Assay (ELISA)

To avoid the possibility that the His tag on the expressed proteins might affect the results, we purified GST-tagged Fim2, FHA_1877–2250_, and PtxS1 to coat the plate for ELISAs. Briefly, The sequences of GST-Fim2, GST-PtxS1 and GST-FHA_1877–2250_ were codon-optimized and synthesized by Detai Biotechnology Co., Ltd. (Nanjing, China), and the following upstream and downstream primers were used to amplify the Fim2-Gst, PtxS1-Gst, and FHA_1877–2250_-Gst fragments from the synthesized sequences: Fim2-Gst-F and Fim2-Gst-R (5′-GCGTCGACGATGATGGTACCATCGTG-3′ and 5′-CGGAATTCCGGATAAACACGCTAAA-3′), PtxS1-Gst-F and PtxS1-Gst-R (5′-GCGTCGACGATGATCCGCCGGCGAC-3′ and 5′-CGGAATTCGAAGCTGTACGCAATGCT-3′), FHA_1877–2250_-Gst-F and FHA_1877–2250_-Gst-R (5′-GCGTCGACGACGAGCACCGTCACCTG-3′ and 5′-CGGAATTCACCATCCGCCAGGCTGGT-3′). The three resulting fragments were each digested with *Sal*I and *Eco*RI and then connected to a correspondingly digested pGEX-4T-1 plasmid to construct three expression vectors (pGEX-4T-1-Fim2, pGEX-4T-1-PtxS1, and pGEX-4T-1-FHA_1877–2250_) that expressed the three GST-tagged proteins. We then used the same purification method as described above to obtain the GST-tagged Fim2, FHA_1877–2250_, and PtxS1 proteins. GST-tagged Fim2, FHA_1877–2250_, and PtxS1 were each diluted in carbonate coating buffer (50 mM Na_2_CO_3_–NaHCO_3_, pH 9.6) to a concentration of 20 µg/mL and used to coat 96-well immunoplates (100 µL/well), which were then incubated at 4 °C overnight. Next, the plates were washed three times with PBST (500 µL Tween-20 was added to 1L PBS buffer) and patted dry before 200 µL of blocking buffer containing 5% milk in PBS were added to each well. The plates were then incubated at 37 °C for 2 h, after which they were washed and patted dry again. Serial dilutions of mouse serum in dilution buffer (PBS supplemented with 0.5% (*w*/*v*) milk) were then added and incubated at 37 °C for 1 h. After another washing and drying step, 1:20,000-diluted HRP-conjugated IgG (Abcam, Shanghai, China) was added as the secondary antibody. The plates were then incubated at 37 °C for 1 h and washed and dried again, before 100 µL of TMB substrate solution was added to each well. Next, the plates were incubated in the dark at room temperature for 15 min for color development before 50 µL of stop solution (2 M H_2_SO_4_) was added. Five minutes after the reaction was terminated, a microplate spectrophotometer was used to measure the absorbance at a wavelength of 450 nm.

### 2.7. In Vitro Serum Bactericidal Assay

*B. pertussis* Bp148 was cultured until it reached an OD_600_ of 2.0 before dilution with physiological saline to obtain a diluted suspension with a concentration of 100–200 colony-forming units (CFU)/10 µL. Next, 10 µL of diluted bacterial suspension was mixed with 10 µL of serially diluted serum (preheated in a 58 °C water bath for 30 min to inactivate complement (Pel-Freez) and 20 µL of complement. After incubation at 37 °C for 1 h, the mixture was coated onto plates containing pertussis charcoal medium and further incubated for 4–5 days at 37 °C. Finally, colony counts were recorded, from which the in vitro serum bactericidal rates were calculated.

### 2.8. Determination of Bacterial Loads in the Lungs and Spleens

A non-lethal dose of 1 × 10^8^ CFU/mouse *B. pertussis* bacterial suspension was injected intraperitoneally on the 14th day after the third immunization. On the seventh day post-infection, mice were sacrificed via neck dissection, and the spleens and lungs were extracted and homogenized with sterile normal saline. The resulting homogenate was diluted to 10^−4^ and 10^−6^ with sterile normal saline. Next, 100 µL of each dilution was coated onto plates containing pertussis charcoal medium and cultured in 37 °C for 4–5 days before bacterial counts were recorded. Finally, the bacterial loads in the spleens and lungs of various groups were calculated.

### 2.9. Histopathological Analysis of Lung Tissues

Lung tissues extracted from immunized mice or normal mice were fixed with 4% paraformaldehyde (Solarbio, Beijing, China) before being paraffin-sectioned and stained with a hematoxylin-eosin (HE) staining kit (Solarbio) used in accordance with the manufacturer’s instructions. In brief, fixed samples were embedded and sectioned before conventional dewaxing and hydration was carried out, after which HE staining of the tissue sections was conducted; afterward, dehydration, cleaning for transparency, and mounting with neutral resin were performed successively. Then we calculated the ratio of alveolar area to lung area in each pathological section to determine the degree of lung injury.

### 2.10. Challenge Experiment

A lethal dose (6 × 10^8^ CFU/mouse) of *B. pertussis* was administered intraperitoneally to mice on day 14 after their last immunization. The mice were then monitored for six days, and the survival rate was recorded.

### 2.11. Statistical Analysis

Antibody titers and bacterial loads were log10-transformed. Statistical analyses were conducted using GraphPad Prism version 8.0 (GraphPad, San Diego, CA, USA). Data are expressed as means ± SD and were analyzed by one-way ANOVA with Dunnett’s multiple-comparison test for the multiple-group comparisons. Differences with a *p*-value of <0.05 were considered significant.

## 3. Results

### 3.1. Protein Expression and Purification

**Figure 1:**
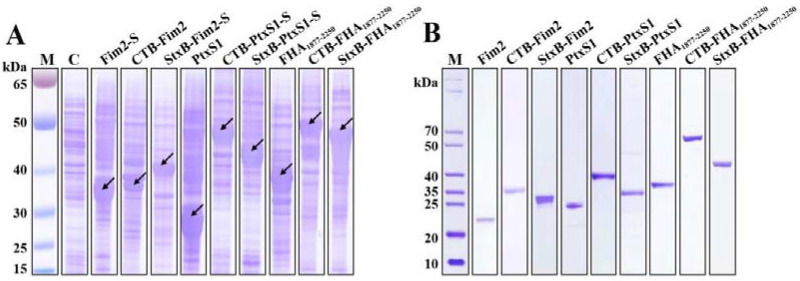
Expression and purification of the recombinant proteins. (**A**) Various recombinant vectors were transformed into *E. coli* BL21, and IPTG was used for induction of protein expression before SDS-PAGE was conducted on cell lysates Coomassie blue staining was performed to analyze the expression of the recombinant proteins. Lane C contains a BL21 strain lacking an expression plasmid that was processed via the same method (negative control). (**B**) The corresponding proteins were purified from the strains with expression plasmids, and the attached SUMO tags (if present) were removed by SUMO-specific proteases. The proteins were then separated by SDS-PAGE and analyzed by Coomassie blue staining.

To enhance the immunogenicity of the pertussis antigens, we first fused CTB or StxB to the N-terminals of Fim2, PtxS1, or FHA_1877–2250_ by constructing pET-CTB-Fim2 and pET-StxB-Fim2-SUMO, pET-CTB-PtxS1-SUMO, pET-StxB-PtxS1-SUMO, and pET-CTB-FHA_1877–2250_ and pET-StxB-FHA_1877–2250_ expression vectors, respectively. Some of these also included a fused 6×His tag and SUMO tag at the C-terminal end to allow their soluble expression. In addition, we also constructed vectors (pET-Fim2-SUMO, pET-PtxS1, and pET-FHA_1877–2250_) to express the three antigens alone. After transformation of these vectors into *Escherichia coli* BL21 cells and IPTG induction, the whole-cell sample of each strain was analyzed by SDS–PAGE and Coomassie blue staining. The results show the detection of high expression bands at approximately 34, 27, and 36 kDa in the antigen-only lanes, which correspond to the molecular weights of Fim2-SUMO (Fim2-S), PtxS1, and FHA_1877–2250_, respectively. Increases in the molecular weights of these fusion protein bands were observed in the lanes containing CTB-Fim2, StxB-Fim2-SUMO (StxB-Fim2-S), CTB-PtxS1-SUMO (CTB-PtxS1-S), StxB-PtxS1-SUMO (StxB-PtxS1-S), CTB-FHA_1877–2250_, and StxB-FHA_1877–2250_, which also corresponded with their expected molecular weights (Figure 1A). Following purification through affinity chromatography and removal of the SUMO tags (if present), Coomassie blue staining were performed, and the purity of the obtained proteins was found to be over 90% (Figure 1B), which met the requirements for use in subsequent animal experiments.

### 3.2. Potency Assays of Serum Antibody

**Figure 2:**
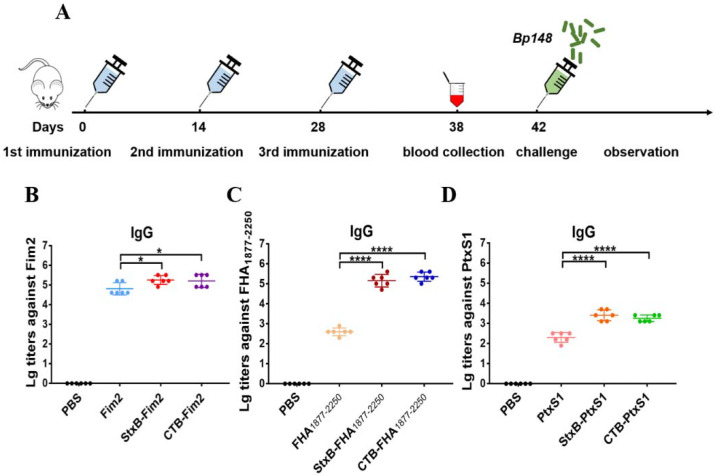
Antigen-specific serum antibody responses in mice after immunization. (**A**) Schematic diagram of the mouse immunization and evaluation experiment. (**B**) Serum IgG antibody titers against Fim2 antigen in mouse groups after their immunization with PBS, Fim2, StxB-Fim2, or CTB-Fim2. (**C**) Serum IgG antibody titers against FHA_1877–2250_ antigen in mouse groups after their immunization with PBS, FHA_1877–2250_, StxB-FHA_1877–2250_, or CTB-FHA_1877–2250_. (**D**) Serum IgG antibody titers against PtxS1 antigen in mouse groups after their immunization with PBS, PtxS1, StxB-PtxS1, or CTB-PtxS1. The serum antibody titer of each mouse was log10-transformated, and the data are expressed as means ± SD. A one-way ANOVA with Dunnett’s multiple-comparison test was used for each multiple-group comparison (**** *p* < 0.0001 and * *p* < 0.05).

After confirming that the endotoxin content of all vaccines including Fim2, StxB-Fim2, CTB-Fim2, PtxS1, StxB-PtxS1, CTB-PtxS1, FHA_1877–2250_, StxB-FHA_1877–2250_, and CTB-FHA_1877–2250_ were at a low level (Figure S2), each of the six purified CTB- or StxB-based fusion antigens and three unfused antigens (2 µg of pertussis antigen/mouse), along with 100 µg aluminum hydroxide adjuvant, were used to immunize five-week-old female BALB/c mice subcutaneously on Days 0, 14, and 28. Blood from the tail veins of mice was collected on Day 10 after the last immunization, and the corresponding serum was isolated for use in an ELISA (Figure 2A). The results show that the various immunization groups each produced high levels of IgG antibody compared with the control group. The highest antibody titers obtained of each of the various groups were around 10^5^. Mice that received CTB- or StxB-Fim2 vaccines had significantly higher Fim2-specific IgG titers compared with Fim2-vaccined mice (Figure 2B). The antibody titers of mice immunized with StxB-FHA_1877–2250_ or CTB-FHA_1877–2250_ were each about 100-fold greater than that in mice immunized with FHA_1877–2250_ (Figure 2C). The serum-specific titers of mice induced by the PtxS1-fusion antigens were also approximately 10-fold higher than that induced by PtxS1 alone (Figure 2D). Together, these results show that CTB- and StxB-based vaccines can significantly increase serum antibody potency over that of antigen alone. Although the antibody against CTB or StxB was also induced (Figure S3), we thought the protection is mainly contributed by the antigen-specific immune response because our previous study has confirmed that immunization of carrier protein alone cannot provide protection against infection in another bacteria [19].

### 3.3. In Vitro Bactericidal Assay of Serum against B. pertussis

**Figure 3:**
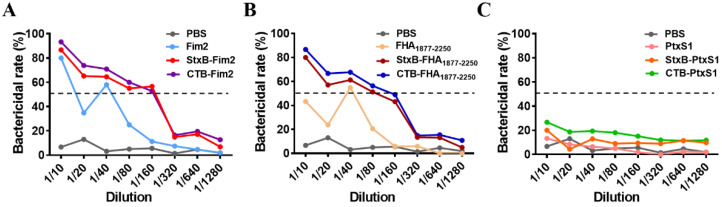
Evaluation of mice serum bactericidal activity. Different dilutions of serum from immunized mice (with inactived complement), *B. pertussis* suspension, and complement were co-incubated and cultured to enumerated colonies. The in vitro serum bactericidal activity was then calculated via comparison with the blank control group (without serum). (**A**) In vitro bactericidal efficiency of different dilutions of serum from mice in the PBS, Fim2, StxB-Fim2, and CTB-Fim2 groups. (**B**) In vitro bactericidal efficiency of different dilutions of serum from mice in the PBS, FHA_1877–2250_, StxB-FHA_1877–2250_, and CTB-FHA_1877–2250_ groups. (**C**) In vitro bactericidal efficiency of different dilutions of serum from mice in the PBS, PtxS1, StxB-PtxS1, and CTB-PtxS1 groups. The dotted lines represent the serum bactericidal rate of 50%.

To further test the bactericidal activity of antibodies, the serum samples were diluted and incubated with *B. pertussis* Bp148 strain and complement (Pel-Freez) in vitro. The results show that the bactericidal efficiencies of serum from mice immunized with either Fim2 or FHA_1877–2250_ fusion antigens were higher than those of mice immunized with the antigens alone. They also reveal higher serum bactericidal titers (defined as the serum dilution when the bactericidal activity is 50%) in mice immunized with CTB- or StxB-fused antigens (Figure 3A,B). Although we found that there was no significant dose–response relationship in the serum bactericidal activity after immunization with PtxS1 antigen or the PtxS1 fusion antigens, the bactericidal rate of all groups was below 30% (Figure 3C); this may be because PT is a secretory toxin whose specific antibodies would likely not recognize the bacteria themselves.

### 3.4. Measurement of Bacterial Loads in Spleens and Lungs

**Figure 4:**
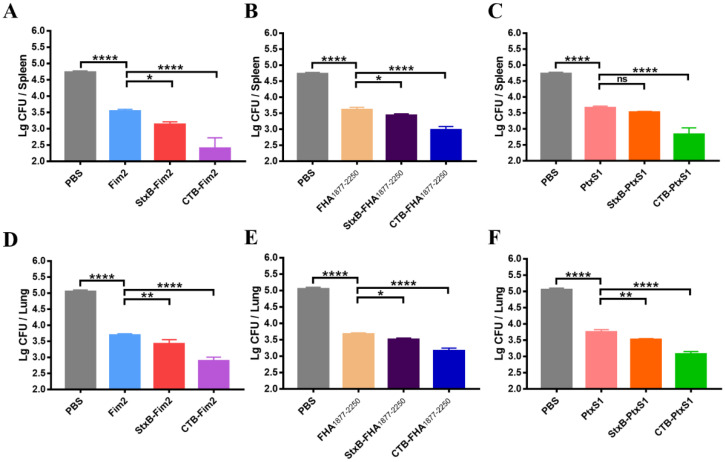
Bacterial loads in the spleens and lungs of immunized mice. (**A**) Bacterial loads in the spleens of mice from the PBS, Fim2, StxB-Fim2, and CTB-Fim2 groups. (**B**) Bacterial loads in the spleens of mice from the PBS, FHA_1877–2250_, StxB-FHA_1877–2250_, and CTB-FHA_1877–2250_ groups. (**C**) Bacterial loads in the spleens of mice from the PBS, PtxS1, StxB-PtxS1, and CTB-PtxS1 groups. (**D**) Bacterial loads in the lungs of mice from the PBS, Fim2, StxB-Fim2, and CTB-Fim2 groups. (**E**) Bacterial loads in the lungs of mice from the PBS, FHA_1877–2250_, StxB-FHA_1877–2250_, and CTB-FHA_1877–2250_ groups. (**F**) Bacterial loads in the lungs of mice from the PBS, PtxS1, StxB-PtxS1, and CTB-PtxS1 groups. Data are expressed as the means ± SD and were analyzed by a one-way ANOVA with Dunnett’s multiple-comparison test for the multiple-group comparisons (* *p* < 0.05, ** *p* < 0.01, **** *p* < 0.0001).

After confirming that the antibodies induced by the CTB- or StxB-based vaccine candidates had efficient in vitro bactericidal activity, we conducted in vivo experiments to examine the protective effects of the vaccine candidates by intraperitoneally injecting mice with a dose of 1 × 10^8^ CFU/mouse of *B. pertussis* after immunization. We found that on Day 7 post-challenge, the bacterial loads in spleens of immunized mice from the Fim2, StxB-Fim2, and CTB-Fim2 groups were significantly lower compared with those of mice from the PBS group, with reductions of 84%, 97%, and 99%, respectively (Figure 4A). The FHA_1877–2250_ and PtxS1 groups had similar results (Figure 4B,C). The bacterial loads in the spleens of mice immunized with antigen alone, StxB-antigen and CTB-antigen were respectively 90%, 94%, and 94% lower than that of mice in the PBS group when FHA_1877–2250_ was used as the antigen. Besides, they were respectively 92%, 95%, and 98% lower when PtxS1 was used as the antigen.

Because the lung is a major target organ for *B. pertussis* infection, we also measured the post-infection pulmonary bacterial load. We found that the Bp148 bacterial load in the lungs from PBS-vaccinated mice reached approximately 10^5^ CFU. Although significant decreases in the bacterial load were observed in the lungs of antigen alone-immunized mice, dramatic further decreases were observed for mice vaccinated with CTB- or StxB-fused antigens. Notably, the bacterial loads of the CTB-Fim2, CTB-FHA_1877–2250_, and CTB-PtxS1 groups were approximately 99%, 99%, and 98%, respectively, lower than that of the PBS group. These findings demonstrate that CTB- or StxB-based vaccines (particularly CTB-based ones) outperform vaccination with antigen alone for inducing the clearance of infectious bacteria from the spleens and lungs.

### 3.5. Histopathology Analysis of Lung Tissues

**Figure 5:**
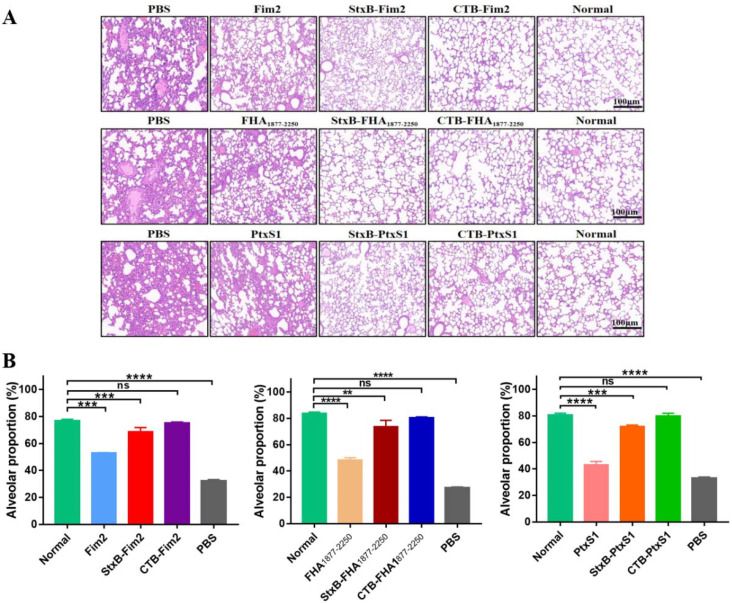
Histopathology of the lung tissues from BALB/c mice after their infection with *B. pertussis*. Lung tissues from the differently immunized mice were separated on Day 7 post-intraperitoneal challenge with a dose of 1 × 10^8^ CFU/mouse *B. pertussis*. Tissues were fixed in 4% paraformaldehyde, embedded in paraffin, dewaxed, sectioned, and finally subjected to HE staining. (**A**) Representative pathological section in different groups. Normal indicates lung tissue from uninfected mice. (**B**) The proportion of alveolus in pathological section were analyzed. Data are expressed as the means ± SD and were analyzed by one-way ANOVA with Dunnett’s multiple-comparison test for the multiple-group comparisons (** *p* < 0.01, *** *p* < 0.001, **** *p* < 0.0001).

Given the success of the CTB- and StxB-based vaccines at increasing bacterial clearance in the lungs of mice, we next applied a previously tested immunization and challenge protocol and then conducted HE staining of lung tissues on Day 7 after the mice were infected with a dose of 1 × 10^8^ CFU/mouse *B. pertussis* to further analyze the protection induced by the vaccines. In line with our bacterial load observations, the HE staining analysis of dissected lungs revealed similar trends in injury and pathology levels for each of the different treatments. Compared with the uninfected mice, the lungs of mice in the PBS group showed severe tissue injury, which mainly presented as decreased alveolar expansion, alveolar structure disruption, lung interstitial expansion and inflammatory cell infiltration, and poor overall pulmonary ventilation. Although pathological damage was alleviated in the lungs from the three groups of mice immunized with antigen alone, the aforementioned severe pathological changes were still present. In contrast, we found that mice immunized with CTB- or StxB- fused antigens, particularly CTB-fused ones, had almost no lung injury after infection (Figure 5A). In addition, we quantitatively analyzed lung damage through the proportion of alveolus, and the results also showed that the CTB- or StxB-based vaccines provide strong protective effects (Figure 5B). Therefore, these findings suggest that—compared with immunization with antigen alone—immunization with CTB- or StxB-based vaccines results in better lung protection after infection with *B. pertussis*.

### 3.6. Protective Immunity against Lethal Challenge with B. pertussis by Vaccination

**Figure 6:**
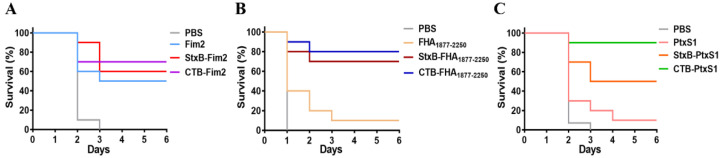
Survival of immunized mice after lethal challenge with *B. pertussis.* At day 14 after the last immunization, all mice were challenged intraperitoneally with a dose of 6 × 10^8^ CFU/mouse *B. pertussis*. Mice were monitored for 6 days, and their survival rates were recorded. (**A**) Survival of mice in the PBS, Fim2, StxB-Fim2, and CTB-Fim2 groups after challenge. (**B**) Survival of mice in the PBS, FHA_1877–2250_, StxB-FHA_1877–2250_, and CTB-FHA_1877–2250_ groups after challenge. (**C**) Survival of mice in the PBS, PtxS1, StxB-PtxS1, and CTB-PtxS1 groups after challenge.

Having confirmed the significantly enhanced immune effect and protection induced by CTB- and StxB-based vaccines during a non-lethal infection in immunized mice, we further assess the protective effects of these vaccines by conducting a lethal dose challenge. The mice were injected intraperitoneally with the higher dose of 6 × 10^8^ CFU/mouse *B. pertussis* on day 14 after their third immunization. The survival of mice was then monitored continuously for 6 days. All mice in the control (PBS-vaccinated) group died within three days after challenge. Regarding Fim2, the survival rate of Fim2 group was 50%, whereas the survival rates for the StxB and CTB fusion protein groups were 60% and 70%, respectively (Figure 6A). The FHA_1877–2250_ antigen alone provided almost no protective effect (10% survival rate) against Bp148, whereas the survival rates in the StxB-FHA_1877–2250_ and CTB-FHA_1877–2250_ groups were 70% and 80%, respectively (Figure 6B). Similarly, the survival rates in the PtxS1 antigen fusion groups were also significantly higher compared with that of the antigen alone group. Specifically, the CTB-PtxS1 group had a survival rate of 90%, whereas that of the StxB-PtxS1 group was only 50% (Figure 6C). These results suggest that vaccination with CTB- or StxB-fused antigens can significantly improve the survival rate of mice after lethal challenge over that of vaccination with antigen alone and that the carrier protein CTB has a stronger effect compared with StxB.

## 4. Discussion

Vaccines are a primary method for preventing bacterial infections. Although the pertussis vaccine has been widely administered to children for more than 50 years, pertussis remains a poorly controlled, vaccine-preventable bacterial disease [20]. The switch from whole cell pertussis vaccines (wPVs) to aPVs was an important turning point in pertussis prevention history. However, aPVs offer far lower immune protection compared with wPVs [4,5]. In addition, strain variation and improvements in diagnostic techniques have resulted in persistently high pertussis incidence and even pertussis outbreaks in many countries and regions in recent years [21,22]. Because pertussis is still a threat to human health, there is an urgent need to produce a safe pertussis vaccine with better protection than those currently available.

Previous studies have assessed the immunogenicity of vaccines containing one or many *B. pertussis* components (such as PT and FHA), but the induced immune responses were not ideal [23]. When recombinant pertussis antigens (rFim2 or rPRN) were used as vaccines for immunization, the survival rate of immunized mice was only 20–40%, even though the antibody potency was high [24]. In the present study, we selected Fim2, FHA_1877–2250_, and PtxS1 as target antigens. Our results show that although immunization with these antigens alone did not provide adequate protection in subsequent protective evaluations, such immunization was able to stimulate an effective immune response with the production of specific antibodies, which is consistent with previous reports. Our study also showed that vaccination with any of these antigens fused to one of the carrier proteins resulted in immune-boosting effects, with higher antibody levels, bacterial clearance rates, and protection from disease as compared with vaccination using a pertussis antigen alone. Therefore, this fusion antigen strategy has great potential for use in generating vaccines to prevent infection.

CTB and StxB are widely used to increase the immunogenicity of antigens because of their unique adjuvant characteristics, lack of toxicity, and stability and ease of use after their fusion with peptides or proteins [25,26]. The increased protection provided by fusion antigens observed in this study is mainly attributed to the fused carrier proteins (CTB and StxB). Both of these carriers belong to the bacterial AB5 toxin family, which can naturally form a pentameric structure and thus provide a more complex structure for the antigen. In addition, when CTB or StxB is used as a carrier for antigen delivery, they will be recognized by their specific cell surface receptors and enhance the uptake of the antigen by antigen-presenting cells, which affects the intensity of the ensuing specific immune response [27]. Our histopathological examinations of lung tissue revealed that CTB- and StxB-based vaccines could both provide effective protection for the lungs. The protection rate induced by vaccination with the CTB- and StxB-fused antigens was also far higher than that induced by vaccination with the antigen alone, particularly for CTB-PtxS1, which conferred 90% protection against lethal challenge, and CTB-FHA_1877–2250_, which provided 80% protection. Therefore, bacterial AB5 toxin-based vaccines hold great promise for use in the development of prophylactic vaccines [27].

In recent years, subunit vaccines have been considered as one of the most promising vaccine types and have become a hotspot of research and development. However, the major shortcoming of subunit vaccines is that their immunogenicity is usually low, so adjuvants and repeated immunizations are typically required [28]. To increase the immunogenicity and stability of subunit vaccines, fusion expression with an appropriate vector is an effective approach. Our research has demonstrated the advantages of using bacterial AB5 toxin protein for enhancing antigen immunity, and previous studies have reported that further design and modification on the basis of AB5 toxin protein can upgrade the vector to nanoscale, thus additionally improving the immune response and protection conferred by the vaccine [29,30]. In addition, some nanocarriers, such as virus-like particles, are increasingly being used in antigen presentation and delivery [31]. Relevant follow-up research on the vaccine candidates described in this study will also be carried out by our group.

## Data Availability

Data is contained within the article or supplementary material.

## Supplementary Materials

The following are available online at https://www.mdpi.com/article/10.3390/vaccines9060542/s1, Table S1: Animal immunization strategy; Figure S1: Amplification of pertussis antigen fragment; Figure S2: Determination of endotoxin content; Figure S3: The antibody titers against CTB or StxB in different groups.
