# Supplementary material for: Expression of Bordetella pertussis Antigens Fused to Different Vectors and Their Effectiveness as Vaccines
Source: Vaccines (Basel). 2021 May 21;9(6):542. doi: 10.3390/vaccines9060542 (PMC8224380; doi:10.3390/vaccines9060542)
Supplement: Supplementary file 1 [file vaccines-09-00542-s001.zip › vaccines-1167171-supplementary.pdf]

Table S1. Animal immunization strategy

Group	Dose (pertussis antigen in each candidate / mouse)	Volume	Adjuvant ($Al(OH)_3$)
Fim2	2 μg	100 μL	100 μg
CTB-Fim2	2 μg		
StxB-Fim2	2 μg		
Control	PBS		
PtxS1	2 μg		
CTB-PtxS1	2 μg		
StxB-PtxS1	2 μg		
Control	PBS		
$FHA_{1877-2250}$	2 μg		
CTB- $FHA_{1877-2250}$	2 μg		
StxB- $FHA_{1877-2250}$	2 μg		
Control	PBS		

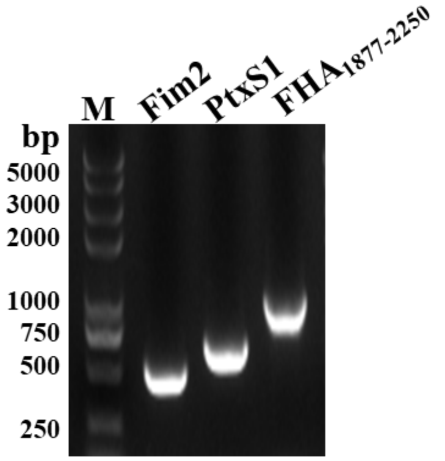

Figure S1. Amplification of pertussis antigen fragment.

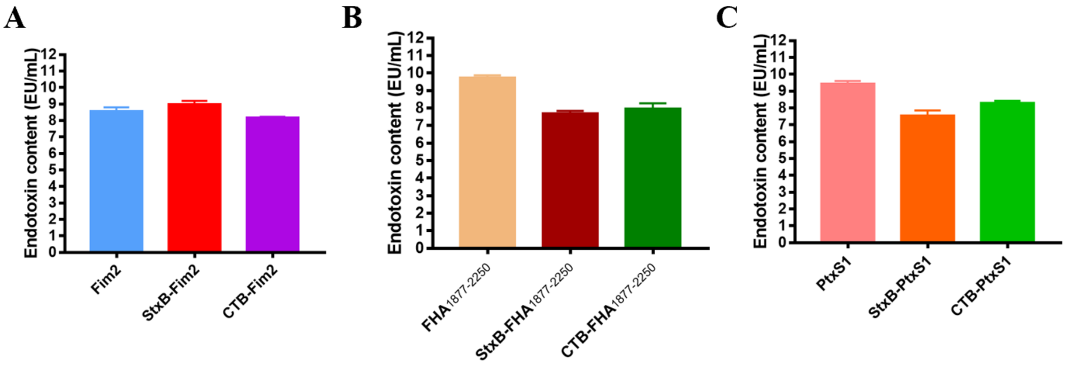

Figure S2. Determination of endotoxin content.

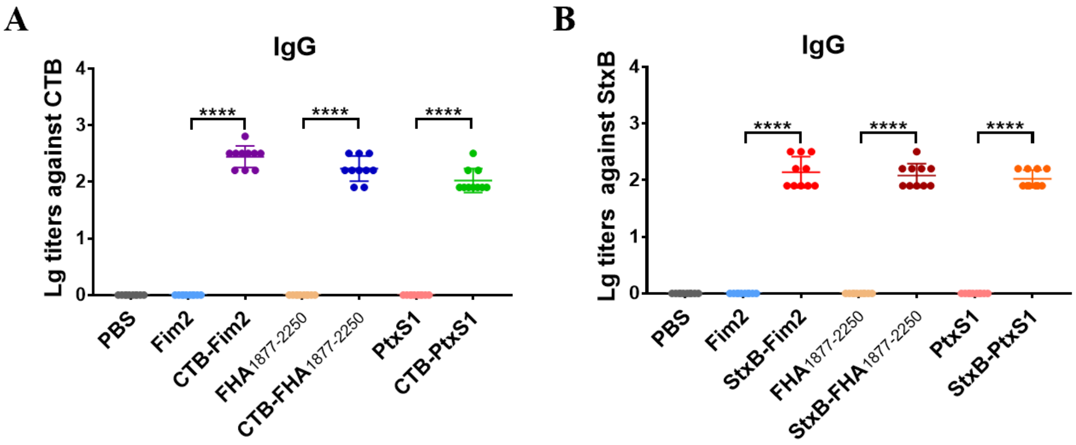

Figure S3. The antibody titers against CTB or StxB in different groups.
